# Multiple Novel Functions of Henipavirus O-glycans: The First O-glycan Functions Identified in the Paramyxovirus Family

**DOI:** 10.1371/journal.ppat.1005445

**Published:** 2016-02-11

**Authors:** Jacquelyn A. Stone, Anthony V. Nicola, Linda G. Baum, Hector C. Aguilar

**Affiliations:** 1 Paul G. Allen School for Global Animal Health, Washington State University, Pullman, Washington, United States of America; 2 Department of Veterinary Microbiology and Pathology, Washington State University, Pullman, Washington, United States of America; 3 Department of Pathology and Laboratory Medicine, University of California, Los Angeles, California, United States of America; Harvard Medical School, UNITED STATES

## Abstract

O-linked glycosylation is a ubiquitous protein modification in organisms belonging to several kingdoms. Both microbial and host protein glycans are used by many pathogens for host invasion and immune evasion, yet little is known about the roles of O-glycans in viral pathogenesis. Reportedly, there is no single function attributed to O-glycans for the significant paramyxovirus family. The paramyxovirus family includes many important pathogens, such as measles, mumps, parainfluenza, metapneumo- and the deadly Henipaviruses Nipah (NiV) and Hendra (HeV) viruses. Paramyxoviral cell entry requires the coordinated actions of two viral membrane glycoproteins: the attachment (HN/H/G) and fusion (F) glycoproteins. O-glycan sites in HeV G were recently identified, facilitating use of the attachment protein of this deadly paramyxovirus as a model to study O-glycan functions. We mutated the identified HeV G O-glycosylation sites and found mutants with altered cell-cell fusion, G conformation, G/F association, viral entry in a pseudotyped viral system, and, quite unexpectedly, pseudotyped viral F protein incorporation and processing phenotypes. These are all important functions of viral glycoproteins. These phenotypes were broadly conserved for equivalent NiV mutants. Thus our results identify multiple novel and pathologically important functions of paramyxoviral O-glycans, paving the way to study O-glycan functions in other paramyxoviruses and enveloped viruses.

## Author Summary

Glycosylation is a protein modification process that occurs inside cells, in which specific types of sugars (glycans) are added to certain amino acids in some proteins. Glycosylation happens for many organisms, from microbes to mammals, including many pathogens. Altered glycosylation is increasingly being associated with auto-immune diseases and cancer, highlighting the need to better understand glycosylation. There are two types of sugars added during the glycosylation process: N-glycans and O-glycans. While the roles of N-glycans have been extensively reported for many organisms, the roles of O-glycans remain largely unknown, particularly for viruses. The paramyxoviruses are a medically important family of viruses that include the highly lethal Hendra (HeV) and Nipah (NiV) viruses. Viral entry into host cells and spread from cell to cell relies on two viral proteins: G and F. Here we mutated known O-glycan locations in the HeV and NiV G proteins. Loss of O-glycans affected several viral processes crucial to viral entry and spread from cell to cell. Our results are the first reported functions for paramyxoviral O-glycans, contributing to the field of viral entry and spread, and helping pave the way for future functional studies in other pathogens.

## Introduction

Many microbial pathogens utilize protein glycosylation for host invasion and immune evasion [1]. Although many N-glycan functions have been reported, relatively little is known about the roles of O-glycans in microbial pathogenesis or biology, particularly for viruses. O-linked glycosylation is a ubiquitous protein modification in organisms belonging to several kingdoms. For example, O-glycans play roles in protein trafficking, signaling, cell-cell interactions, and receptor binding for host proteins [2–4], and O-glycans are important for developmental processes and immune system functions [3]. Additionally, altered O-glycosylation has been linked to illnesses such as autoimmune diseases and cancer [5–7], as well as pathogen virulence [8–10]. Yet currently the specific functions of O-glycans on viral glycoproteins are not well understood, and to our knowledge there is no single function attributed to O-glycans for the important paramyxovirus family.

Conversely, viral glycoprotein N-glycans are known to be critical for proper protein folding and trafficking, receptor interactions, cell adhesion, and evasion of host immune responses [reviewed in [1]]. In addition, loss of paramyxoviral N-glycans reduces or increases membrane fusion capacity and processing of the F glycoproteins of measles virus (MeV), NiV, and Sendai virus [11–13], and N glycans on NiV F and G modulate membrane fusion and viral infectivity, and protect the virus from antibody neutralization [12,14–17]. Additionally, galectin-1, a lectin that binds specific N-glycans and O-glycans, inhibits NiV cell-cell fusion when added post-infection, but can enhance viral entry into endothelial cells by increasing viral attachment to target cells [18–20].

While potential N-glycosylation sites are marked by a distinctive N-X-S/T motif (where N is asparagine, X is any residue except proline, S is serine, and T is threonine), the determinants that cause addition of O-glycans to S or T residues are incompletely understood. Moreover, O-glycosylation of one S or T can affect O-glycosylation of other S or T residues [21]. O-glycans can act as an antibody shield for the gammaherpesvirus bovine herpes virus (BoHV-4) [22], affect binding of herpes simplex virus 1 (HSV1) gB to the paired immunoglobulin-like type 2 receptor alpha (PILRα) receptor and hence PILRα-dependent viral entry [23,24], and O-glycans on HSV1 gC-1 are thought to affect receptor binding and cell-cell spread [25]. For HSV-2, viral O-glycans have been recently shown to stimulate an antiviral immune response upstream from interferons [26]. Additionally, an O-glycan-rich region of Ebola virus GP plays a role in cell detachment [27], though this function has yet to be linked directly to the O-glycans themselves. O-glycans are present on the human immunodeficiency virus (HIV) and simian immunodeficiency virus (SIV) envelope, Marburg spike protein, mouse hepatitis virus (MHV) E1 protein, and vaccinia hemagglutinin (HA) [28–32], but the functions of these O-glycans are unknown. For the paramyxoviruses, it is known that O-glycans are *not* involved in respiratory syncytial virus (RSV) G oligomerization [33] *nor* Newcastle disease virus (NDV) receptor binding [34]. To our knowledge, however, no study has reported any functions for paramyxoviral O-glycans.

The *Paramyxoviridae* family is comprised of negative-sense single-stranded enveloped RNA viruses including measles (MeV), respiratory syncytial (RSV), Newcastle disease (NDV), parainfluenza (PIV), metapneumo- (MPV), and the deadly Henipaviruses (HNV) NiV and HeV among others [35]. HeV and NiV are biosafety level 4 (BSL4) pathogens with no approved vaccine or treatment for humans. Individuals infected with NiV or HeV suffer from respiratory distress and encephalitis, with 40–90% mortality rates [36]. A hallmark of HNV viral infections is extensive formation of syncytia (cell-cell fusion). These viruses can be transmitted from animal-to-animal, animal-to-human and human-to-human [37], highlighting a need for understanding the mechanism of HNV spread between individuals and within infected individuals.

**Fig 1:**
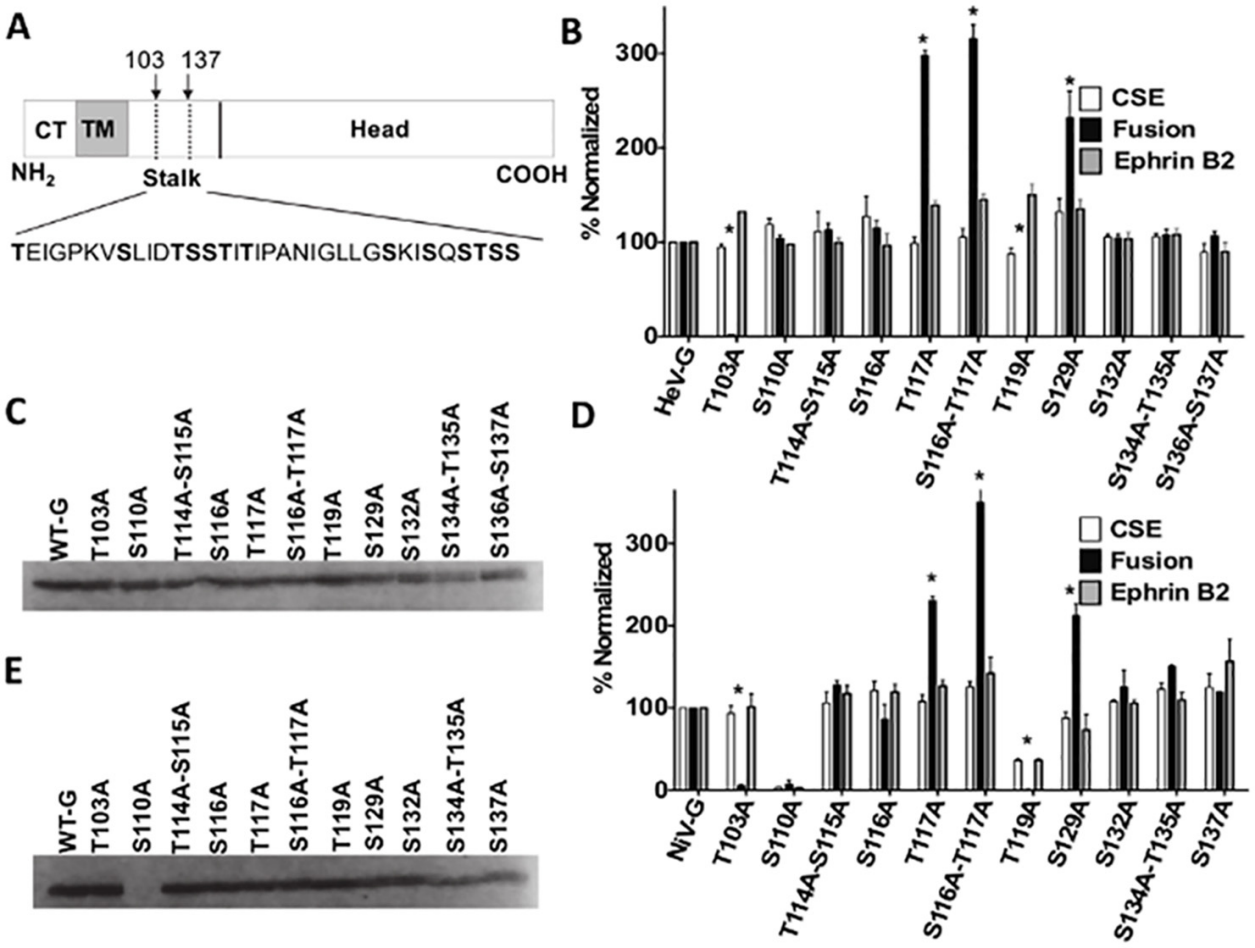
O-glycans on the HNV G stalk modulate fusion. A) Schematic of the HNV G protein. The dashed lines denote the O-glycan rich region in which mutants were created, with the insert showing the amino acid sequence of that region. Amino acids determined by mass spectrometry to be O-glycosylated in HeV are in bold. CT = cytoplasmic tail, TM = transmembrane. B&D) Cell surface expression (CSE, white) fusion (black), and ephrinB2 binding (grey) levels of generated HeV G (B) or NiV G (D) mutants. All values were normalized to wt HeV or NiV G. Averages and standard deviations are shown. N = 5. Statistically significant differences, as determined by a Student’s t-test, are marked with an * (p< 0.05). C, E) Western blot analysis of cell lysates from HeV G (C) or NiV G (E) mutants.

Paramyxoviruses use two transmembrane glycoproteins to facilitate both viral-cell fusion during viral entry, and the pathognomonic syncytia formation. The attachment (H, G, or HN) protein binds the cell receptor, in turn activating the fusion protein (F) to insert its hydrophobic fusion peptide into the host cell membrane and execute viral-cell membrane fusion, resulting in viral entry into the host cell [38]. Via a similar mechanism, F and H/G/HN also cause cell-cell fusion of infected-healthy cells. For HNV, the attachment protein G binds the cell receptor ephrinB2 or ephrinB3 [39–41]. HNV G consists of an N-terminal cytoplasmic tail followed by a transmembrane region, a stalk domain, and a C-terminal globular head (Fig 1A), responsible for receptor binding [42].

The H/G/HN stalk plays an important role in paramyxovirus-induced membrane fusion, executed by the F glycoprotein. Disrupting the H/G/HN stalk inhibits fusion [43–45], and headless mutants are capable of causing fusion, underlining that the stalk itself is key in the fusion triggering process [46–49]. Recently, multiple specific O-glycan structures in a stalk domain of HeV G were identified [50], but the functions of these O-glycans are unknown. Since the HeV G stalk plays an important role in viral-cell and cell-cell membrane fusion, and O-glycan structural data is available for HeV, we used HeV G as a model to investigate the functions of O-glycans in henipaviral and paramyxoviral biology.

We mutated each O-glycosylated Ser or Thr in the heavily O-glycosylated HeV G stalk domain 103–137 to alanine (Ala), to prevent O-glycosylation at specific sites on the protein. Loss of these O-glycans affected membrane fusion levels, resulting in both hyper- and hypo-fusogenic phenotypes. Multiple mutants displayed altered HeV G/F association, viral entry capabilities in a pseudotyped virus system, and F incorporation into pseudotyped virions, and one mutant had an altered conformation and altered receptor-induced conformational changes important for membrane fusion. Furthermore, equivalent mutants in NiV G displayed highly-conserved roles, with most mutants replicating the HeV phenotypes, although some distinct photypes were observed for NiV, suggesting overall high conservation of O-glycan functions for this genus. This study is the first to uncover multiple functions of O-glycans in the paramyxovirus family, identifies the highest number of functions of O-glycans for any single virus, and points to differences between N- and O-glycan functions for the paramyxoviruses.

## Results

### O-glycans in the HNV G stalk domain modulate membrane fusion

The specific locations and structures of O-glycans in HeV G have been identified via mass spectrometry [50], and except for one, all were found in the stalk. We mutated each O-glycosylated S or T residue in the heavily O-glycosylated stalk domain 103–137 to an alanine (A) to prevent O-glycosylation at each specific site (Fig 1A). Multiple adjacent O-glycosylation sites were mutated simultaneously to limit the number of target mutants, thus we initially constructed nine mutants. The S116A-T117A double mutant was then split into two single mutants, S116A and T117A, respectively, for a total of eleven mutants.

We first tested the ability of the mutant HeV G proteins to induce cell-cell fusion. For consistency, we used 293T cells to provide a similar cell background as that used for the HeV G O-glycan structural studies by Colgrave *et*. *al*. [50]. 293T cells were transfected with wild-type (wt) HeV F and either wt HeV G or an O-glycan mutant. After 18–24 hours post-transfection, nuclei in syncytia (fused cells) were quantified microscopically, with a syncytium defined as four or more nuclei within a single cell membrane, and compared to the wt G [49]. Interestingly, five of the eleven mutants showed differences in cell-cell fusion, with reduced fusion levels for T103A and T119A (2% and 0%, respectively) and increased fusion for T117A, S116-T117A, and S129A (232–316%). These mutants are the first to show that viral O-glycans can have an effect on modulating the extent of cell-cell fusion (Fig 1B). Interestingly the single mutant S116A did not yield an altered fusion phenotype, suggesting that the hyper-fusogenicity of the S116A-T117A double mutant is largely due to the S117A substitution (Fig 1B).

We then used flow cytometry to ask if these fusion phenotypes were due to altered cell surface expression (CSE) levels of the mutant HeV G proteins. 293T cells transfected with either wt or mutant G plasmids were collected 18–24 hours post-transfection and stained with a 1° antibody to the extracellular C-terminal hemagglutinin (HA) tag in the proteins, to avoid false conformational influences on the mutant’s real CSE levels, followed by a fluorescent 2° antibody. CSE of mutant HeV G proteins were normalized to the values of wt HeV G. All mutants were expressed at the cell surface at levels comparable to that of wt HeV G (87–115%, Fig 1B). Normalized fusion levels were then divided by normalized CSE values to obtain a fusion index, with hyperfusogenic mutants defined as having a fusion index ≥1.5, and hypofusogenic mutants having a fusion index ≤0.5 (Table 1) [14]. All mutants also bound soluble B2-hFC as detected by flow cytometry at levels similar to their respective CSE values (Fig 1B), which is expected since the G head, not the stalk, is responsible for ephrinB2 binding [42,51]. These results suggest that the mutants’ altered membrane fusion capabilities were not due to differences in CSE or receptor binding. In addition, Western blot analysis using an anti-HA antibody confirmed that all mutants were expressed in cell lysates (Fig 1C).

**Table 1:** Fusion indices of HeV and NiV O-glycan mutants. Normalized fusion values were divided by normalized CSE values for each mutant. As values for fusion and CSE for the WT proteins were by definition set at 100%, the WT fusion index was set as 1. Hyperfusogenic mutants are defined as ≥1.5, and hypofusogenic mutants are defined as ≤0.5. Hyper and hypo-fusogenic mutants are in bold. Averages are shown. Statistical analysis was performed using a Student’s t-test. Statistically significant differences are marked with an * (p<0.05).

Mutant	CSE/Fusion (HeV)	CSE/Fusion (NiV)
WTG	1	1
**T103A**	0.02*	0.06*
S110A	0.87	-
T114A-S115A	1.01	1.20
S116A	0.90	0.72
**T117A**	2.99*	2.14*
**S116A-T117A**	3.02*	2.79*
**T119A**	**0**	**0**
**S129A**	1.76*	2.43*
S132A	0.99	1.16
S134A-T135A	1.02	1.23
S136A-S137A	1.01	1.04

The stalk domains of HeV and NiV are highly conserved, with 90% amino acid identity between the two. After observing that loss of O-glycans in HeV G affected cell-cell fusion, we created equivalent mutants in NiV G. Thus we generated the same eleven NiV G mutants, with the exception of the HeV G double mutant S136A-S137A, which became a single mutant S137A in NiV, as residue 136 in wt NiV is already an alanine. Remarkably, we observed very similar overall fusion phenotypes for NiV, with the T103A and T119A mutants being hypo-fusogenic (6% and 0% fusion, respectively) and T117A, S116A-T117A, and S129A being hyper-fusogenic (212–350% fusion). CSE levels for these mutants were similar to HeV as well, with the exception that T119A expressed at the cell surface at only ~36% in NiV compared to ~87% in HeV, and S110A did not express at all for NiV (Fig 1D). Fusion indices for the NiV G mutants showed similar patterns to HeV as well (Table 1). All NiV G mutants bound soluble B2-hFC at levels similar to their CSE levels (Fig 1D). Western blot analysis of the NiV G mutants showed that, again, all with the exception of S110A were expressing within cell lysates (Fig 1E), including T119A, which was expressed poorly at the cell surface, suggesting that T119A’s reduced CSE may be due to an alteration in trafficking to the cell surface. Altogether these results are the first demonstration of a role for O-glycans in modulating paramyxovirus-induced membrane fusion, and suggest this function is highly conserved among the henipaviruses.

### G/F association is altered by O-glycan loss, but does not determine membrane fusogenicity

**Fig 2:**
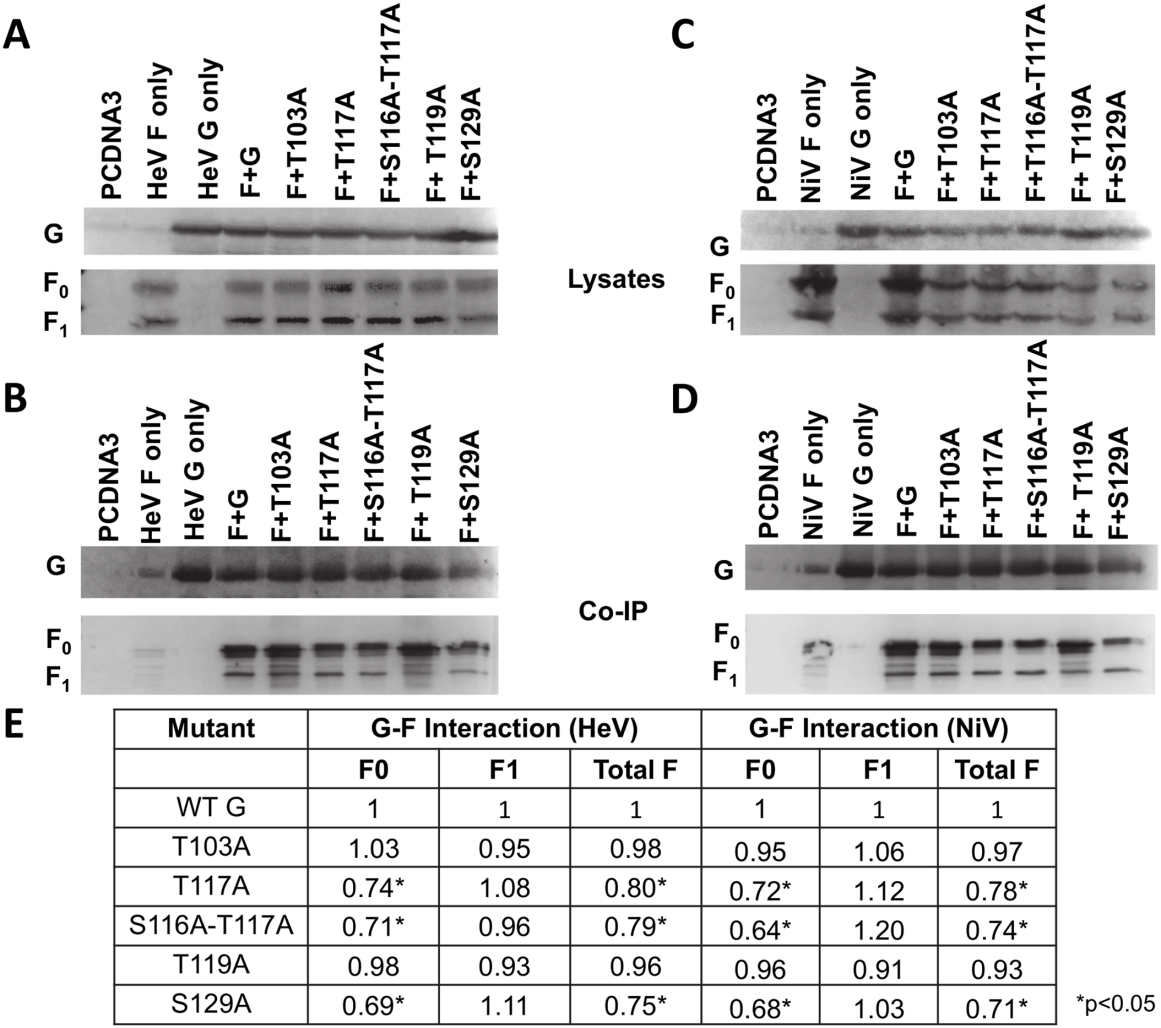
Associations with F are altered by O-glycan loss. A) Cell lysates of cells transfected with wt F and either wt or mutant HeV G. HeV G (top) is blotted with rabbit anti-HA, and HeV F (bottom) is blotted with mouse anti-AU1. B) Pulldown of HeV G and co-IP of F for transfected cells. Antibody blotting was performed as in A). C) Cell lysates of cells transfected with wt F and wt or mutant NiV G. D) Co-IP of NiV transfected cells. E) Co-IP values of HeV and NiV O-glycan mutants, determined by densitometry. Image Lab software (Biorad) was used to measure the densitometry of Western blot bands. Co-IP values were determined by dividing either the F_0_, F_1_, or total F densitometry values for the co-IP bands by the co-IP G densitometry values to account for any differences in co-IP G pulldown. These values were then divided by F_0_, F_1_, or total F densitometry values for cell lysates to account for differences in F mutant expression. Values were then normalized to those of wt HNV G. Averages are shown. N = 3. Statistically significant differences, as determined by a Student’s t-test, are marked with an * (p<0.05).

Though the specifics of the HNV F and G spatiotemporal interactions are still largely unknown, the available evidence suggests that HNV F and G interact on the cell or viral membrane prior to receptor binding, after which F and G dissociate from each other, triggering F to execute fusion (dissociation model) [38,52,53]. We tested the interactions of the HeV G and NiV G O-glycan mutants with HeV or NiV F, respectively, through a co-immunoprecipitation (co-IP) assay. 293T cells were co-transfected with wt HNV F and wt or mutant HNV G. After 16–20 hours cells were collected in lysis buffer and applied to a co-IP column (Miltenyi Biotec). Cell lysates and co-IP eluates were separated by SDS-PAGE and blotted using rabbit anti-HA (to detect G) and mouse anti-AU1 (to detect F) 1° antibodies, followed by fluorescent 2° antibodies. HNV F is produced as an uncleaved precursor, F_0_, that is cleaved by cathepsin L upon endocytosis into F_1_ and F_2_ subunits, linked by a disulfide bond [54,55]. The F_0_ (precursor) and F_1_ (processed) bands are both AU1-tagged and thus visible in Western blots, resulting in two bands for F and one for G (Fig 2). Co-IP values calculated via quantitative Western blot analyses (Fig 2E) revealed that the hyper-fusogenic mutants all had decreased F_0_ association (0.64–0.74 co-IP values), though this decrease in association was not observed for the F_1_ band, suggesting that removing O-glycans from these residues may alter intracellular F/G interactions, and to a lesser extent or not at all surface interactions (Fig 2). The nature of co-immunoprecipitation assays to study membrane protein interactions, including the use of detergents and the artificial association of membrane proteins via micelle formation, may account for the lack of differences in G/F_1_ interactions for the mutants, as further discussed in our Discussion section. Thus, even though the G/F1 interactions do not seem to be affected, it is possible that the altered F_0_/G interactions suggest overall effects in the G/F interactions, which generally support the dissociation model for HNV. These results were consistent for both the HeV G and NiV G mutants. Most likely, however, our G/F_1_ interaction data is consistent with the hyper- or hypo-fusogenic phenotypes of the O-glycan mutants not being a result of the effects of the O-glycan mutants on F/G interactions.

### Loss of specific O-glycans can affect HNV G conformations

**Fig 3:**
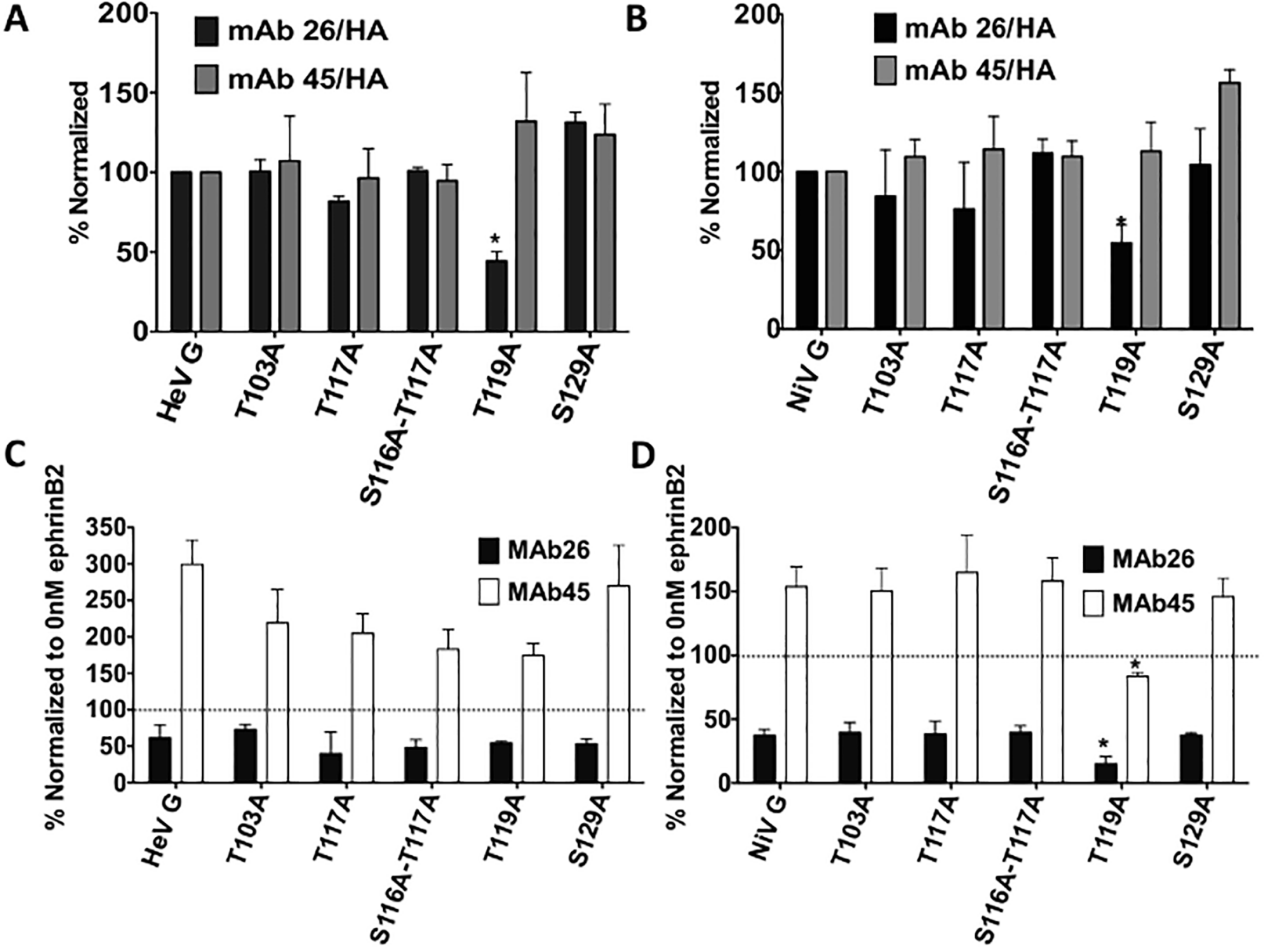
O-glycan loss can affect HNV G conformation. A & B) Normalized mAb binding to wt or mutant HeV G (A) or NiV G (B). All antibody binding levels are normalized to the CSE of each mutant (as measured by HA levels). N = 3. C & D) Conformational changes of wt or mutant HeV G (C) or NiV G (D) as measured by Mab45 (white) and Mab26 (black) upon the addition of 100nM ephrinB2. All values are normalized to antibody binding levels when no (0nM) ephrinB2 was added. Averages and standard deviations are shown. N = 3. Statistically significant differences, as determined by a Student’s t-test, are marked with an asterisk (p<0.05).

It is possible that loss of O-glycans could affect glycoprotein conformations, so we next tested if differing conformations correlated with the altered fusion phenotypes we observed. We have previously generated conformational antibodies to NiV G, some of which also bind HeV G. The first, Mab45, binds at the base of the head near the stalk, while the second, Mab26, binds the top of the head near the receptor binding site of both NiV and HeV [49,56]. We tested the binding of these antibodies to HNV G via flow cytometry and found that, while the majority of the mutants had antibody binding profiles similar to those of the wt HNV G, the fusion dead T119A mutant showed decreased Mab26 binding, suggesting that this mutant has an altered conformation for both viruses (Fig 3A and 3B).

Mab45 and Mab26 can also measure receptor-induced conformational changes in HNV G that are important to trigger F. We previously showed that upon receptor binding a conformational change occurs in NiV G region 9 (bound by Mab26), followed by a conformational change in region 4 (bound by Mab45), leading to uncovering of a specific domain at the C-terminus of the stalk, which triggers F. Moreover, mutants unable to fully undergo these conformational changes were hypo-fusogenic, demonstrating that these conformational changes in G are important for fusion [49]. These conformational changes can be measured by comparing antibody binding in the absence vs. presence of soluble ephrinB2 receptor binding. With no receptor present, Mab26 binding to G is relatively higher, but decreases as more ephrinB2 is added. Mab45 exhibits the opposite trend, with binding of Mab45 to G increasing upon the addition of soluble ephrinB2 [49].

Thus we tested Mab26 and Mab45 binding to wt or mutant HNV G at 0nM and 100nM ephrinB2. 100nM values were normalized to those at 0nM to represent the amount of increased or decreased antibody binding in the presence of receptor. All HeV mutants showed a decrease in Mab26 binding and an increase in Mab45 binding to some degree, suggesting the mutants are all still able to make the conformational changes involved in fusion (Fig 3C). Interestingly however, when the same assays were performed with the NiV G mutants, the T119A mutant showed decreased Mab45 and Mab26 binding in 100nM ephrinB2 compared to wt G (Fig 3D), suggesting that the hypo-fusogenicity of this mutant may in part be due to an inability to undergo the full range of conformational changes required for fusion. These data, combined with T119A’s decreased Mab26 binding in the absence of receptor, suggest that this mutant is in a near post-receptor binding conformation, preventing it from undergoing the Mab45 binding enhancement indicative of a full-range conformational switch from pre to post receptor binding conformations.

In addition, all mutants co-migrated with wt HNV G when analyzed by *semi-denaturing* SDS PAGE, with all relative levels of tetramers, dimers, and monomers not significantly affected by the loss of an O-glycan as determined by densitometry (S1 Fig), suggesting that O-glycan loss does not affect G oligomerization. This is important as proper oligomerization is critical for glycoprotein function, and the G stalk is an important determinant of G oligomerization [16,45]. Overall, the majority of the O-glycan mutants’ fusion phenotypes were not due to aberrant G conformations or abilities to undergo receptor-induced conformational changes, with the exception of mutant T119A.

### Loss of O-glycans on HNV G can affect HNV F virion incorporation and viral entry in a pseudotyped viral system

As we found that loss of O-glycans modulated cell-cell fusion, we next tested if this affected viral entry, using a validated BSL2 pseudotyped virus infection assay [14]. Vesicular stomatitis virus (VSV) virions containing a *Renilla* luciferase gene in place of the VSV glycoprotein G gene, and expressing HNV wt F with either wt or mutant HNV G on the outer membranes were created as described previously [14]. These pseudotyped virions were tested for viral entry using a *Renilla* luciferase reporter assay. Vero cells were infected with the pseudotyped virions and incubated for 2 hours at 37°C. Growth media was then added, and at 18–24 hours post infection the cells were collected, lysed, and luminescence was determined. Relative light units (RLU), representing viral entry for each of the mutants, were compared to those of wt HNV G. Western blot analysis of pseudotyped virion lysates were also performed to assess protein incorporation levels.

**Fig 4:**
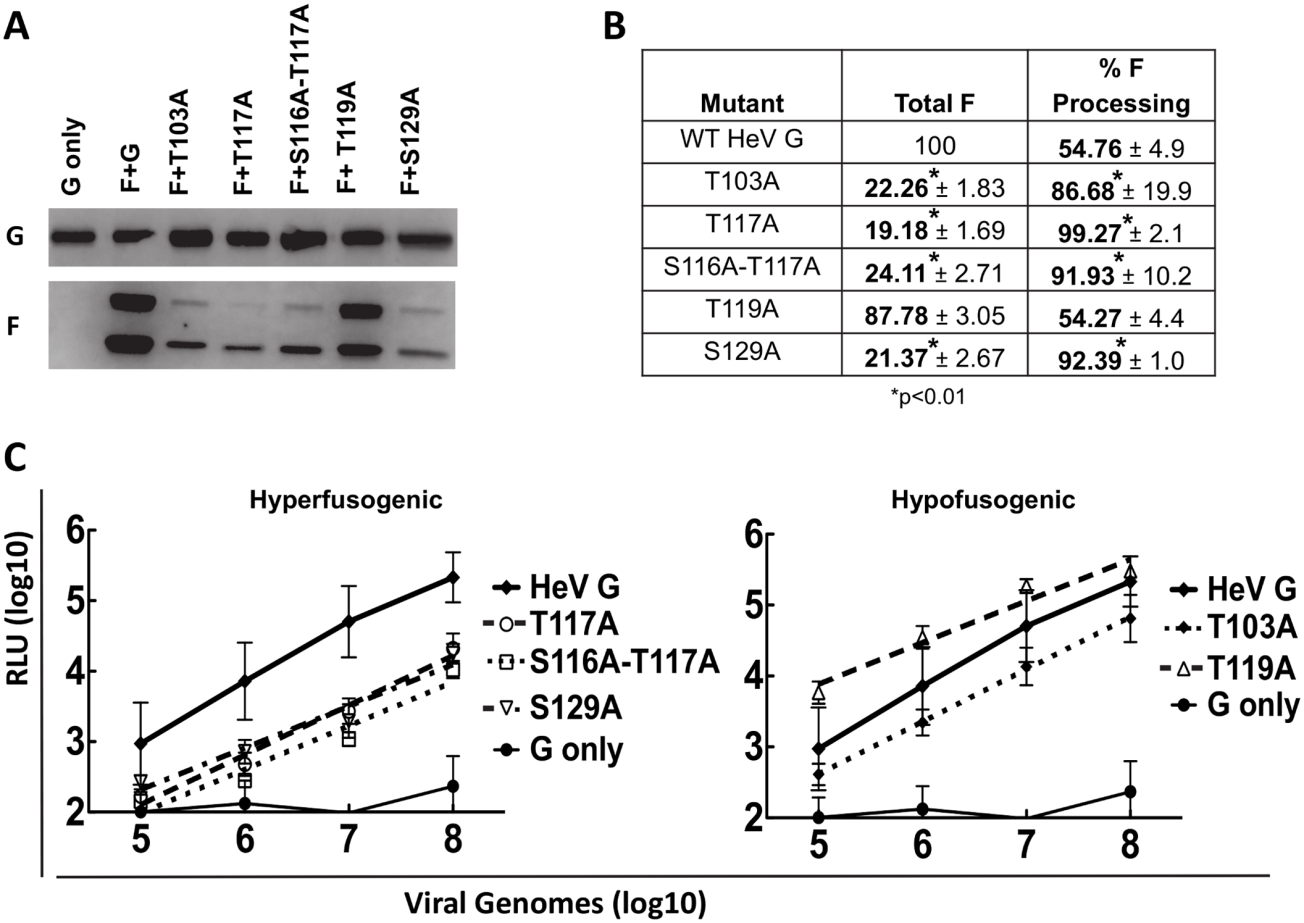
Loss of O-glycans in HeV G affect HeV F incorporation and processing in pseudotyped virions, affecting viral entry. A) Western blot of HeV/VSV viral lysates. HeV G (top panel) and HeV F (bottom panel) were detected as described in Fig 2. B) F incorporation and F processing of the HeV mutant pseudotyped virions determined by densitometry. F incorporation values were normalized to those of wt HeV. F processing was measured by the ratio of F_1_/ (F_0_+F_1_) for HeV pseudotyped virions. N = 3. Statistically significant differences, as determined by a Student’s t-test, are marked with an * (p<0.01). C) Viral entry of HeV/VSV virions at log dilutions of viral input. Values for the mutant virions are compared to wt HeV values. Virions expressing only HeV G (no HeV F) were used as a negative control. Averages and standard deviations are shown. N = 3.

Remarkably, Western blot analysis showed that, although G incorporation for each of the HeV G mutants was similar to wt G, F incorporation was reduced and F processing was enhanced for all G mutants except T119A (Fig 4A). Densitometry confirmed this observation; all mutants except for T119A incorporated HeV F at levels only 19–24% of that observed for wt pseudotyped virions and increased processing of F, as determined by the ratio of F_1_/total F, which was 86–99% for the mutants as compared to 54% for wt pseudotype virions (Fig 4B). Viral entry assays showed that all G mutants displayed reduced entry levels as compared to wt G, once again with the exception of T119A (Fig 4C), even though we would expect the three hyper-fusogenic mutants to enter at levels higher than that of wt HeV G. Reduced pseudotyped virion HeV F incorporation levels likely contribute to the decreased viral entry levels. To our knowledge, this is the first report of a paramyxovirus HN, H, or G mutant affecting F incorporation into virions and thus viral entry, or affecting F processing. It is possible that loss of an O-glycan therefore affects intracellular F/G interactions and/or glycoprotein trafficking, as well as F processing, consistent with our co-IP results (Fig 2). It is noteworthy that the effect of G O-glycan mutants on F processing was not observed in cell lysates (Fig 2). Additionally, flow cytometry using a variety of anti-F antibodies revealed no significant difference in F cell surface expression in the presence of HeV or NiV G (S2 Fig), a finding that also suggests that the altered interactions observed by co-IP (Fig 2) were not due to differences in F cell surface expression.

**Fig 5:**
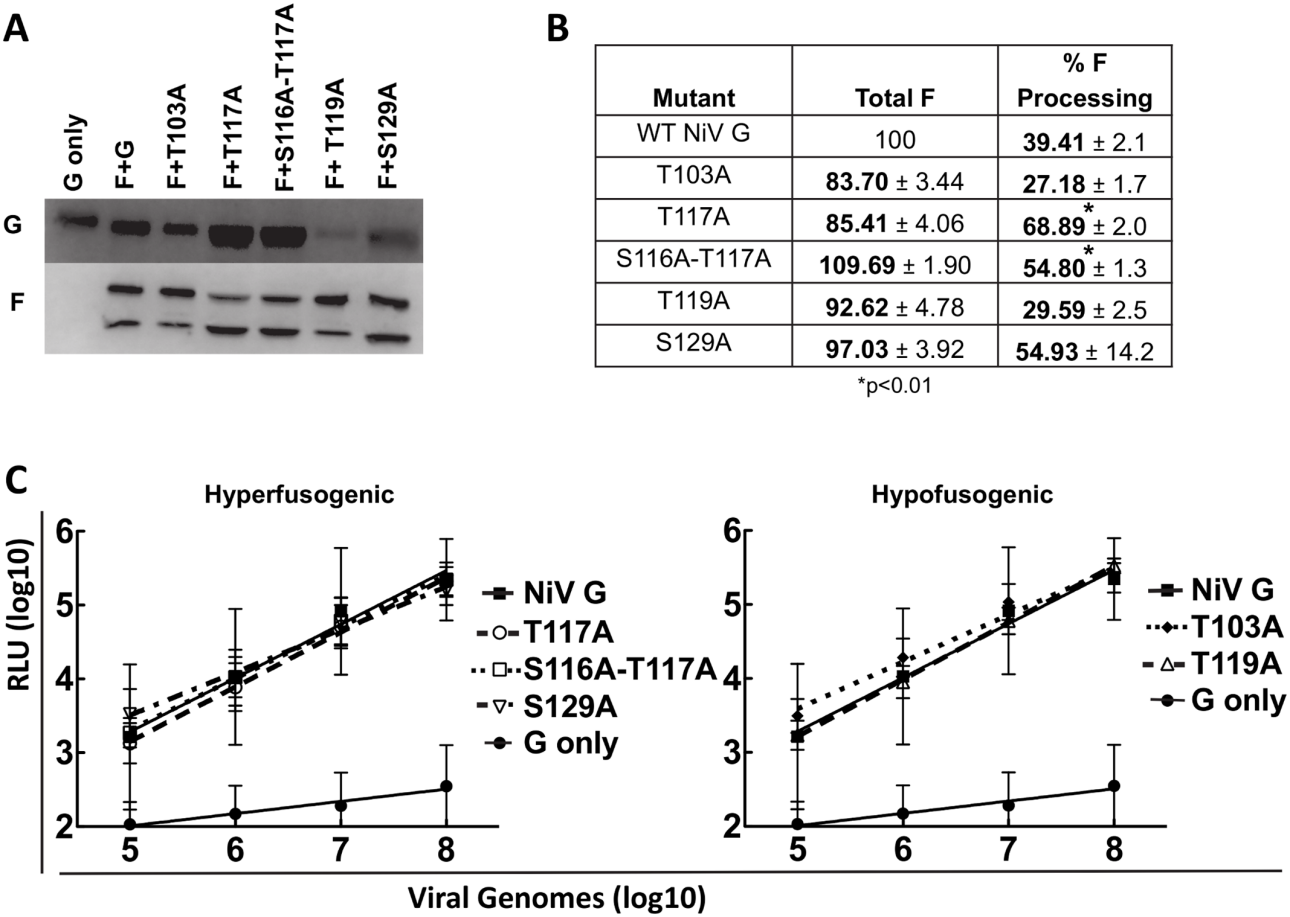
Loss of O-glycans in NiV G does not affect NiV F incorporation in pseudotyped virions, but affects NiV F processing and pseudotyped viral entry. A) Western blot of NiV/VSV viral lysates. NiV G (top panel) and NiV F (bottom panel) were detected as described in Fig 2. B) F incorporation and F processing of the NiV mutant pseudotyped virions determined by densitometry. F incorporation values were normalized to those of wt NiV. F processing was measured by the ratio of F_1_/ (F_0_+F_1_) for NiV pseudotyped virions. N = 3. Statistically significant differences, as determined by a Student's t-test, are marked with an * (p<0.01). C) Viral entry of NiV/VSV virions at log dilutions of viral input. Values for the mutant virions are compared to wt NiV values. Pseudotyped virions expressing only NiV G (no NiV F) were used as a negative control. Averages and standard deviations are shown. N = 3.

Interestingly, Western blot and viral entry assays of the NiV pseudotyped virions did not reveal the same phenotypes. All pseudotyped virions had similar levels of NiV F as assessed by Western blot analysis (Fig 5A) and by densitometry (Fig 5B), and with the exception of T119A, which had less G incorporation, had viral entry values similar to that observed for wt G (Fig 5C). F processing, however, was still altered (Fig 5A and 5B). The hyper-fusogenic mutants T117A, S116A-T117A, and S129A all had higher processing (F_1_) levels (69%, 55%, and 55%, respectively), whereas the wt (39%) and hypo-fusogenic mutants T103A and T119A had lower (27% and 29%) processing (F_1_) levels (Fig 5B). These data further corroborate that HNV G O-glycan loss can affect HNV F in pseudotyped virions, and implies that the mechanisms may differ between HeV and NiV, since F incorporation was affected more for HeV than for NiV. Since equivalent mutant NiV pseudotyped virions are able to enter cells at wt levels with proper F incorporation, the data from NiV pseudotyped virions also further supports the explanation that reduced F incorporation is responsible for the reduced pseudotyped viral entry levels observed in the HeV mutants, at least to some degree. Other potential mechanisms, such as the contribution of O-glycans to the stability of virions, are further explored in the Discussion. Interestingly, the S129A mutant also had less G incorporation (Fig 5A), though it was still able to enter cells at levels similar to wt G, suggesting that with full G incorporation this mutant may have had entry levels greater than the wt G pseudotyped virions.

In summary, we have found that HNV O-glycans had roles in cell-cell fusion, G/F interactions, G conformation, pseudotyped viral entry, and F incorporation and processing in pseudotyped virions. These important viral glycoprotein phenotypes were mostly conserved among HeV and NiV, but some important differences were observed between NiV and HeV O-glycan functions.

## Discussion

Viral O-glycans remain understudied, and the present study is the first to show functions for paramyxoviral O-glycans. In mammalian cells, O-glycans have functions in receptor binding, cell-cell interactions, cellular trafficking, and immune system regulation [2–4]. Viruses use glycosylation for protein trafficking, signaling, folding, immune evasion, and receptor interactions [1], but the majority of these functions have been described for N-glycans. The lack of clear O-glycosylation prediction motifs, and the fact that utilization of some O-glycan sites can affect O-glycosylation of neighboring sites [21], makes analysis of specific O-glycan functions much more complex. O-glycans are present on HIV, SIV, Marburg, MHV, and vaccinia virus glycoproteins [28–32] but their functions are still unknown. While some studies have shown that O-glycans are involved in immune evasion, receptor binding, plaque formation, and possibly cell detachment [22,25,27], this has not been described for paramyxoviruses, although prior work has shown that O-glycans *do not* play roles in RSV-G oligomerization [33] *nor* NDV receptor binding [34]. The present work is the first to show functions for paramyxoviral O-glycans, and one of only a handful of studies demonstrating functions of O-glycans for any virus.

Interestingly, with one exception, all the O-glycans identified in HeV were found in the G stalk domain [50]. Though it is still unclear which regions of HNV G are necessary for fusion, there is strong evidence that the HNV G stalk plays a central role in F triggering. Headless mutants can trigger fusion in PIV5, MeV, NDV, MuV, and NiV [46–49], and disruption of the stalk using various methods perturbs fusion in NiV, MeV, PIV5, and NDV [43–45,57–60]. Here we demonstrated that loss of O-glycans from the stalk of a paramyxovirus attachment protein affects fusion, and in all cases but one, this was not due to altered CSE or receptor binding levels (Fig 1B and 1D). Interestingly, previous studies showed that removal of certain *N-glycans* in HNV F or the G head resulted in hyper-fusogenicity [12,14–16], while removal of one N-glycan from the G stalk resulted in hypo-fusogenicity [15,16], without affecting receptor binding. Our study demonstrates that O-glycans in the stalk are capable of both up- or down-modulating fusion.

There are currently two different interaction models for the two paramyxovirus glycoproteins. In the association model proposed for PIV5 and NDV, for example, F and HN do not interact at the cell surface until receptor binding occurs, after which HN/F association occurs and F is triggered. In contrast, in the dissociation model proposed for MeV, NiV, and HeV, H/F or G/F interact prior to receptor binding; after which the glycoproteins dissociate from each other, triggering F and allowing fusion [38,52]. Reportedly, for HNV hyper-fusogenic and hypo-fusogenic mutants have shown decreased or increased F association, respectively, while receptor binding resulted in partial G/F dissociation [14,56,61], findings that support the dissociation model. Our co-IP data further support this dissociation model for HNV. The hyper-fusogenic mutants had decreased F interactions with F_0_, though this decrease did not extend to the F_1_ subunit (Fig 2), suggesting that the altered interactions may only be with the immature F protein. These results present the possibility that the O-glycans themselves are directly involved in the association between G and F, though these altered interactions are unlikely to account completely, if at all, for the observed fusion phenotypes since G/mature F (F_1_) interactions were not significantly altered. Additionally, the lack of difference in G/F_1_ association between mutants suggests that fusion phenotypes may be independent of F/G association levels. It is also possible, however, that G/F_1_ interactions are altered but not detectable via co-IP. Since co-IP relies on the use of detergents that may alter protein conformations and therefore interactions, results obtained by co-IP are not entirely conclusive. In addition, co-IP can’t distinguish pre vs post-fusion forms of the F protein. Our results, therefore, present the possibility that the observed fusion phenotypes are independent of F/G associations, but it is also possible that differences in mature F/G surface interactions are too small or transient to be observed by co-IP. New methodologies may be needed assess such interaction differences.

Mutation of a single O-glycan addition site in the majority of mutants did not alter protein conformation nor the conformational change capabilities necessary for fusion triggering [49]. The exception was mutant T119A, for which both HeV and NiV G bound Mab26, which detects a pre-receptor binding conformation, at reduced levels (Fig 3). This same mutant in NiV also failed to show enhancement of Mab45 binding upon addition of ephrinB2, suggesting that this mutant may already be in a post-receptor binding conformation at least partially, contributing to the loss-of-fusion phenotype. These data also suggest that retention of G in a pre-receptor binding conformation is important for its F-triggering capability. The HeV T119A mutant still showed enhanced, albeit less than wt HeV G, Mab45 binding after ephrinB2 addition, suggesting that, while HeV T119A may have an altered conformation, implied by decreased Mab26 binding, this conformational change may not be as functionally detrimental as that of NiV T119A. Even though HeV and NiV G are highly homologous, this hints at conformational differences between the two G glycoproteins. This may also relate to the differing CSE levels of these two mutant proteins (87% for HeV and 36% for NiV T119A) (Fig 1B and 1D). It is also noteworthy that loss of an O-glycan did not affect oligomerization for any of these mutants, an interesting finding since O-glycans are in the stalk domain of HNV G, which is crucial for G oligomerization [45].

Although cell-based assays, such as cell-cell fusion, CSE, ephrinB2 binding, F interactions, and conformational change capabilities, yielded similar results for HeV and NiV, we observed more marked differences in pseudotyped virion infections when the O-glycan mutant G proteins were expressed. The NiV G mutant virions all displayed pseudotyped viral entry of Vero cells similar to that observed for wt NiV G (Fig 5C), yet most HeV G mutant pseudotyped virions showed reduced viral entry (Fig 4C). Additionally, pseudotyped virions with mutant HeV G had reduced HeV F incorporation and altered F processing, evidenced by little to no F_0_ present in the viral lysates (Fig 4A and 4B). It is remarkable that loss of O-glycans from HeV G affects F incorporation and processing, and therefore viral entry. Since F may be a driver of viral budding [62], this finding has implications not only for fusion triggering but also for viral assembly and budding, viral particle release, and therefore viral infectivity. O-glycans have been shown to play roles in protein-protein interactions for non-viral proteins [4]. Therefore, it is possible that loss of G O-glycans affects intracellular G/F interactions, thus affecting viral incorporation of both glycoproteins into pseudotyped virions. This is particularly interesting because HeV G and F have been shown to traffic to the cell surface at different rates [63]. Further investigation is needed to determine exactly how mutations in HeV G have such a pronounced effect on HeV F incorporation, particularly in pseudotyped virions, and why this same phenomenon was not observed for NiV G.

Unlike their HeV counterparts, NiV mutant pseudotyped virions incorporated NiV F and also entered cells at wt levels (Fig 5A and 5B), yet F processing was still altered. The hyper-fusogenic mutants, especially T117A and S116A-T117A, showed more efficient F processing (Fig 5A and 5B). Since F_1_ is the mature form of F, more efficient F processing may induce hyper-fusogenicity for these mutants. Since F must be re-internalized for processing and cleavage, loss of specific O-glycans could affect endocytosis of F [54,55]. The hyperfusogenic mutants, for example, showed less interaction with F_0_ via co-IP (Fig 2), which may enable F endocytosis for cleavage into F_1_ and F_2_ more easily than the wt G or the hypo-fusogenic mutants with stronger F interactions, rationalizing the increase in F processing in the hyperfusogenic mutant virions. Our results demonstrating that HNV G mutants affect F processing are unprecedented and unexpected, warranting further mechanistic studies.

Furthermore, premature F triggering on viral surfaces may have also occurred with the HNV pseudotyped virions. Even though the NiV hyper-fusogenic pseudotyped virions entered cells at wt levels (Fig 5C), we would expect increased entry for the hyper-fusogenic mutants compared to wt G. Premature F triggering has been observed and offered as an explanation for differences between cell-cell fusion and pseudotyped viral entry levels for a headless NiV G mutant [49]. As virions are incubated at 37°C in cell supernatants prior to collection, F and G have the opportunity to interact prematurely, triggering F before a target membrane is present. The prematurely triggered F is then unable to trigger fusion once the virions attach to target cells. Since our co-IP results show that hyper-fusogenic mutants already have a lowered association with F, potentially releasing F more readily for fusion, this may explain why the hyper-fusogenic mutant virions would be most affected by premature F triggering. Supporting this notion, pseudotyped virions produced at 32°C instead of 37°C to hinder premature F triggering regained F incorporation and viral entry levels similar to those of the wt pseudotyped virions (S3 Fig), though we acknowledge that there are other potential cellular factors that may have been altered at the lower temperature.

It should be noted, however, that here we have used a pseudotyped viral system instead of actual henipaviruses. While this pseudotype system has been used extensively in previous studies [14–16,49,64], there are limitations to this approach. Though the pseudotyped virions are expressing the HNV glycoproteins on the viral membrane, they still contain the VSV genome and other VSV proteins. The presence of the VSV matrix protein in place of the HNV matrix protein, a protein known to affect viral processes such as budding [65], may alter virion characteristics. It is also possible that interactions occur between the VSV matrix protein and HNV F/G, since it has been suggested that the HNV matrix protein may interact with each of these glycoproteins [62,66]. The viral morphology of the pseudotyped virions is that of the bullet-shaped VSV, instead of the pleomorphic shaped paramyxoviruses, a factor that may also affect viral particle formation and entry. Thus it is possible that the phenotypes observed in the pseudotyped NiV/VSV and HeV/VSV systems would not translate 100% to actual HNV virions. Due to the BSL4 and reverse genetics requirements to assess these differences with actual HeV or NiV, however, the pseudotyped viral system is an effective surrogate system that provides valuable insight, and further BSL4/reverse genetics studies are warranted.

Both N and O-glycans have been shown to shield viruses against antibody neutralization. O-glycans play a role in shielding the gammaherpesvirus BoHV-4 [22], while loss of specific HeV and NiV N-glycans increased virus susceptibility to antibody neutralization [14–16]. Based on these findings, we hypothesized that loss of O-glycans from HNV G would enhance antibody neutralization sensitivity. Antibody neutralization studies, however, showed no antibody neutralization shielding effects (S4 Fig), highlighting a difference in function between N and O-glycans at least for HNV. It is possible that this functional difference is due to the difference in glycan size, as O-glycans are typically smaller than N-glycans [67]. It is also possible that loss of all O-glycans simultaneously, as performed for BoHV-4 [22] would result in increased antibody neutralization susceptibility, but that loss of a single (or two) O-glycan alone is insufficient to have this effect. Alternatively, this part of the G stalk may not be critical for HNV antibody neutralization.

Since it is known that O-glycosylation at one site can affect the glycosylation of neighboring sites [21], mass spectrometry would be needed to determine if loss of an O-glycan at one site affects addition of O-glycans at other sites for our set of O-glycan mutants. Although these studies require high levels of purified recombinant viral glycoproteins, glycobiology expertise, and time of analysis (likely a 1–2 year project), as evidenced by the scarcity of O-glycan studies currently published for viral glycoproteins, this would be a worthwhile future study. The functions of O-glycans observed in the present study, however, are accurate regardless of whether or not neighboring O-glycan sites are affected by loss of O-glycans at our specific mutated sites.

In summary, we have identified multiple novel functions of paramyxovirus O-glycans in HNV, including strong modulation of cell-cell fusion, and effects on G/F interactions, G conformation, receptor-induced G conformational changes, and quite remarkably, F processing and F incorporation into pseudotyped virions. These novel O-glycan functions shed light on the paramyxovirus-induced membrane fusion mechanisms, reveal a new function for G in F interactions, incorporation into virions, and processing, and contribute to our limited knowledge of the functions of viral O-glycans. It remains to be determined if the high densities of O-glycans and their multiple functions on the stalks of the attachment glycoproteins are conserved among all paramyxovirus genera. Moreover, differences in host cell glycosylation machinery may affect the extent of O-glycosylation or O-glycan structures, so that virions produced in different types of host cells may be differentially glycosylated. The current work paves the way for future functional studies of O-glycans for other paramyxoviruses and other viral families.

## Materials and Methods

### Expression plasmids

Codon optimized HNV G and HNV F genes tagged with HA or AU1 tags, respectively, were expressed in PCDNA3.1 or PCAGGS plasmids as previously described [18]. Alanine substitution mutants were created by site-directed mutagenesis of HNV G using a QuikChange kit (Stratagene). Mutations were confirmed by sequencing the entire open reading frame.

### Cell culture

293T (ATCC) and PK13 (ATCC) cells were cultured in Dulbecco’s modified Eagle’s medium with 10% fetal bovine serum (FBS). Vero (ATCC) cells were cultured in minimal essential medium alpha with 10% FBS.

### Cell surface expression, ephrinB2 and antibody binding by flow cytometry

Production of rabbit anti-NiV G monoclonal antibodies has been previously described [56]. Binding of rabbit monoclonal antibodies, mouse anti-HA, or soluble B2-hFc to HNV G wild type (wt) or mutants was measured by flow cytometry. 293T cells were transfected with 2 μg wt or mutant HNV G expression plasmids and collected 20–24 hours post-transfection. Collected cells were incubated 1 hour at 4°C with 1° antibodies diluted 1:100 to 1:1000 and washed three times in FACS buffer (1% FBS with PBS). Cells were next incubated with fluorescent anti-human, anti-mouse, or anti-rabbit Alexa Fluor 647 or 488 antibodies (Life Technologies, NY) diluted 1:200 for 30 min at 4°C, followed by an additional two washes. Cells were fixed in 0.5% PFA and read on a flow cytometer (Guava easyCyte8 HT, EMD Millipore, MA). B2-hFc was used at a 100 μM concentration. For conformational change assays PK13 cells were transfected with wt or mutant HNV G expression plasmids. 0 or 100 μM soluble ephrinB2 was added and cells were incubated for 15 min at 4°C. Mab45 or 26 was added at concentrations specified above and cells were incubated 1 hour at 37°C. 2° antibody, fixation, and cytometer reading were as listed above [49,56].

### Cell-cell fusion quantification

293T cells grown in 6 well plates were transfected at 70–90% confluency with HNV F and either HNV G or G mutant expression plasmids (1:1 ratio, 2 μg total DNA) using BioT or turbofectamine transfection reagent. 18 hours post-transfection cells were fixed in 0.5% paraformaldehyde and syncytia counts were performed under the microscope (200x), with a syncytium being defined as four or more nuclei within a single cell. Five fields per well were counted [14,49].

### Western blot analysis

Transfected cells or pseudotyped virions expressing HNV G wt or mutants with or without HNV F were lysed in RIPA buffer (Millipore) supplemented with complete protease inhibitor (cOmplete Mini, Roche). Lysates were heated at 65°C for 10 minutes and run on a 10% gel for SDS-PAGE analysis. Immunoblots were performed using mouse anti-AU1 or rabbit anti-HA at 1:250 to 1:2000 dilutions. Fluorescent 2° antibodies were diluted 1:1000–1:2000 and blots were imaged on a Li-Cor Odyssey fluorimager.

### Co-immunoprecipitation

Cells transfected with HNV wt or mutant G expression plasmids were lysed in lysis buffer (Miltenyi Biotec). Cell lysates were incubated with 40ul of anti-HA microbeads at 4°C for 30 minutes with rotation. Lysates were purified and eluted over μ columns (Miltenyi Biotec). PAGE was used for cell lysates and column elutions using a 10% gel. F and G proteins were detected by Western blot analysis, as detailed above.

### Pseudotyped virus production and quantification

Pseudotyped virions containing HNV F with wt or mutant HNV G were manufactured as previously described [14,39]. Briefly, 15cm plates of 293T cells were transfected at 37°C with HNV F and wt or mutant HNV G expression plasmids at a 1:1 ratio. After 8 hours the transfection media was switched to growth media. After an additional 16 hours the cells were infected with recombinant VSV-ΔG-rLuc. 2 hours later the infection media was removed and replaced with growth media. 24 hours after infection virions were harvested from cell supernatants using ultracentrifugation, resuspended in NTE with 5% sucrose, and stored at -80°C. Viral RNA was extracted using a QIAamp viral RNA mini kit (Qiagen, CA) and the resulting viral RNA was reverse transcribed using a SuperScriptIII First-Strand Synthesis System for RT-PCR (Invitrogen, NY). Quantitative PCR (qPCR) was performed using a Taqman probe for the VSV genome to quantify viral copy number. For virions produced at 32°C, all transfections and viral infections were carried out at 37°C, and plates were switched to 32°C upon addition of new growth media.

### Pseudotype viral infection assays

Vero cells were infected with tenfold dilutions of pseudotyped virus particles in infection buffer (PBS + 1% FBS) and incubated for 2 hours at 37°C. After 2 hours growth media was added. 18–24 hours after infection cells were lysed and an Infinite M100 microplate reader (Tecan Ltd) was used to measure luciferase activity.

### Antibody neutralization assays

Pseudotyped virions were incubated for 20 min in infection buffer (PBS + 1% FBS) in the presence of varying dilutions (10^-2^ to 10^-7^) of anti HNV G polyclonal antibody. The virus/antibody mixture was added to Vero cells and incubated for 2 hours, after which growth media was added and the cells were incubated an additional 18–24 hours. Viral infection was then measured as described above.

## Supporting Information

S1 Fig Loss of O-glycans does not affect oligomerization. Semi-denaturing SDS-PAGE Western blots of A) HeV and B) NiV cell lysates. Tetramers, dimers, and monomers are noted. C&D) Percentages of tetramers, dimers, or monomers of HeV G (C) or NiV G (D) mutants, as measured by densitometry. N = 3. No statistical differences were detected from wt G (p>0.05).
(TIF)

S2 Fig Cell surface expression of HNV F is not affected by HNV G expression. Cell surface expression values of A) HeV F and B) NiV F in the presence of G, as measured by flow cytometry. 834 and 835 are polyclonal antibodies that bind both HeV and NiV F. 2–36 is an anti-HeV F specific monoclonal antibody. HNV G values were measured using an HA tag. All values are normalized to wt HeV or NiV F or G. Averages and standard deviations are shown. N = 5.
(TIF)

S3 Fig Mutant pseudotyped virions produced at 32°C infected cells at levels similar to wt HNV G. Infection levels of HeV/VSV (top) and NiV/VSV (bottom) virions produced at 32°C at log dilutions of viral input. Virions expressing only HNV G (no HNV F) were used as a negative control. Averages and standard deviations are shown. N = 3.
(TIF)

S4 Fig O-glycans do not affect antibody neutralization levels. Neutralization of A) pseudotyped HeV/VSV or B) pseudotyped NiV/VSV virions at log dilutions of antibody. A panel of four different polyclonal antibodies was used. Neutralization curves of one representative polyclonal antibody per virus are shown, with averages and standard deviations. N = 3.
(TIF)
