# Supplementary figures and images for: Multiple Novel Functions of Henipavirus O-glycans: The First O-glycan Functions Identified in the Paramyxovirus Family
Source: PLoS Pathog. 2016 Feb 11;12(2):e1005445. doi: 10.1371/journal.ppat.1005445 (PMC4750917; doi:10.1371/journal.ppat.1005445)

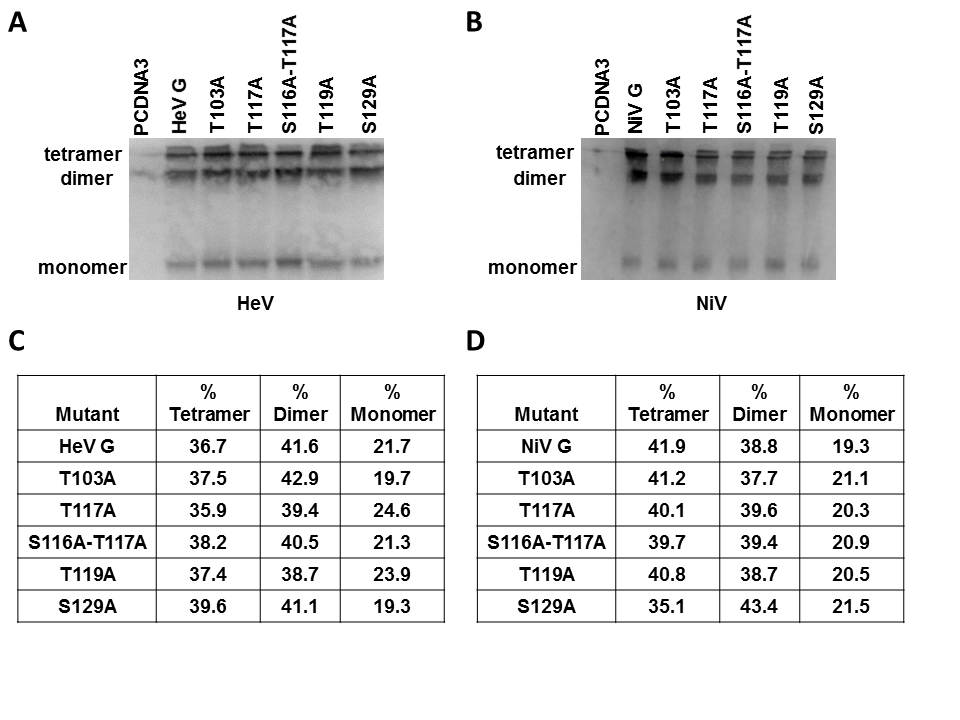

Supplement: S1 Fig — Semi-denaturing SDS-PAGE Western blots of A) HeV and B) NiV cell lysates. Tetramers, dimers, and monomers are noted. C&D) Percentages of tetramers, dimers, or monomers of HeV G (C) or NiV G (D) mutants, as measured by densitometry. N = 3. No statistical differences were detected from wt G (p>0.05). (TIF) [file ppat.1005445.s001.TIF]

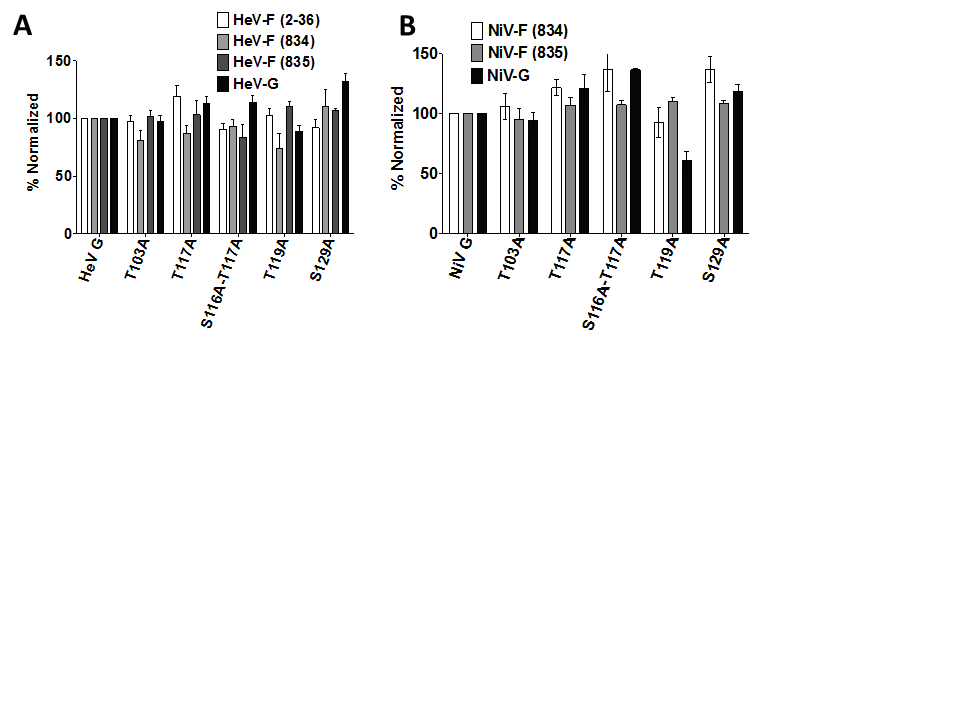

Supplement: S2 Fig — Cell surface expression values of A) HeV F and B) NiV F in the presence of G, as measured by flow cytometry. 834 and 835 are polyclonal antibodies that bind both HeV and NiV F. 2–36 is an anti-HeV F specific monoclonal antibody. HNV G values were measured using an HA tag. All values are normalized to wt HeV or NiV F or G. Averages and standard deviations are shown. N = 5. (TIF) [file ppat.1005445.s002.TIF]

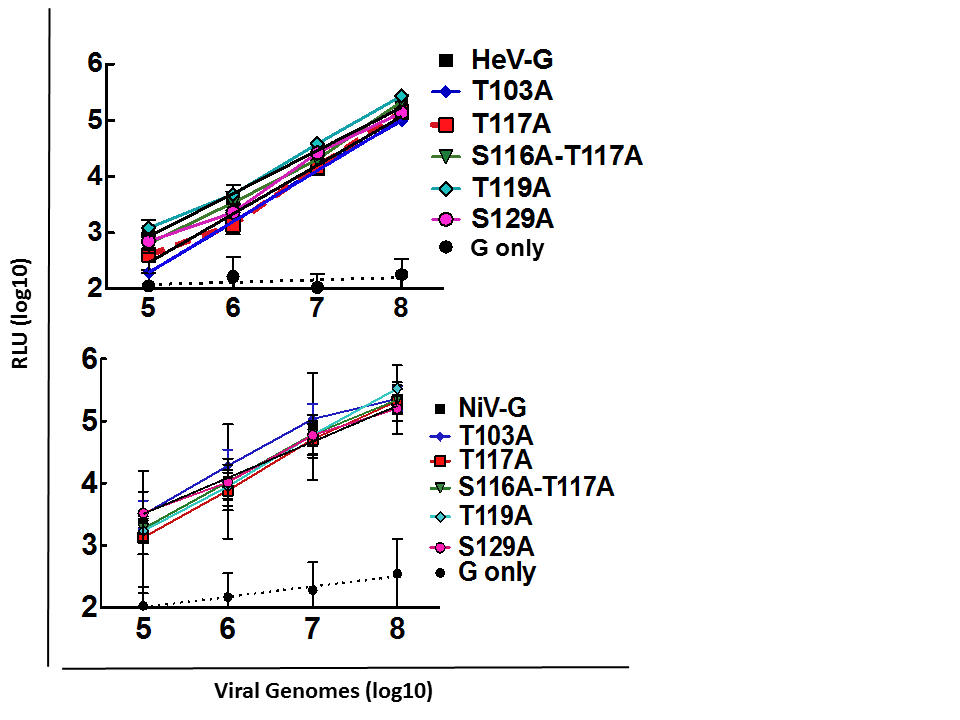

Supplement: S3 Fig — Infection levels of HeV/VSV (top) and NiV/VSV (bottom) virions produced at 32°C at log dilutions of viral input. Virions expressing only HNV G (no HNV F) were used as a negative control. Averages and standard deviations are shown. N = 3. (TIF) [file ppat.1005445.s003.TIF]

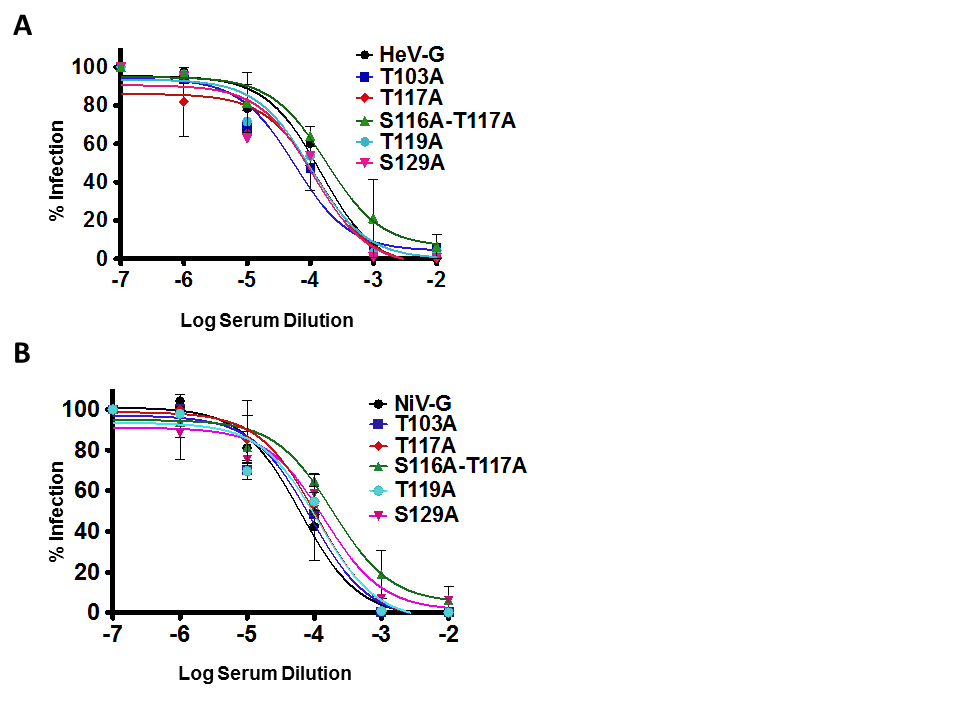

Supplement: S4 Fig — Neutralization of A) pseudotyped HeV/VSV or B) pseudotyped NiV/VSV virions at log dilutions of antibody. A panel of four different polyclonal antibodies was used. Neutralization curves of one representative polyclonal antibody per virus are shown, with averages and standard deviations. N = 3. (TIF) [file ppat.1005445.s004.TIF]
